# Vital information matching in vision-and-language navigation

**DOI:** 10.3389/fnbot.2022.1035921

**Published:** 2022-11-17

**Authors:** Zixi Jia, Kai Yu, Jingyu Ru, Sikai Yang, Sonya Coleman

**Affiliations:** ^1^Faculty of Robot Science and Engineering, Northeastern University, Shenyang, China; ^2^School of Computing, Engineering and Intelligent Systems, Ulster University, Coleraine, United Kingdom

**Keywords:** vision-and-language navigation, multimodal matching, self-tuning module, collaborative learning, vital information matching networks

## Abstract

With the rapid development of artificial intelligence technology, many researchers have begun to focus on visual language navigation, which is one of the most important tasks in multi-modal machine learning. The focus of this multi-modal field is how to fuse multiple inputs, which is crucial for the integrated feedback of intrinsic information. However, the existing models are only implemented through simple data augmentation or expansion, and are obviously far from being able to tap the intrinsic relationship between modalities. In this paper, to overcome these challenges, a novel multi-modal matching feedback self-tuning model is proposed, which is a novel neural network called Vital Information Matching Feedback Self-tuning Network (VIM-Net). Our VIM-Net network is mainly composed of two matching feedback modules, a visual matching feedback module (V-mat) and a trajectory matching feedback module (T-mat). Specifically, V-mat matches the target information of visual recognition with the entity information extracted by the command; T-mat matches the serialized trajectory feature with the direction of movement of the command. Ablation experiments and comparative experiments are conducted on the proposed model using the Matterport3D simulator and the Room-to-Room (R2R) benchmark datasets, and the final navigation effect is shown in detail. The results prove that the model proposed in this paper is indeed effective on the task.

## 1. Introduction

Scholars have pointed out that the core of vision and language navigation task depends on the degree of question answering and executive ability of the machine. In short, the intelligent robot can understand and process visual information and language information, and can better integrate the two types of information and plan the best navigation action, and reach to the target result at last. To obtain better fusion information, it requires the model to understand both the image information, captured in the real-time camera perspective, and the natural language instructions. Then, according to the image information, the agent will acquire its accurate position under the environment of the real world, and follow the action instructions from model decision based on knowledge reasoning to approach to the destination. In this regard, for indoor navigation, Anderson et al. (2018) first proposed a sequence-to-sequence (Seq2Seq) neural network learning model, which outputs one pair of action sequence from two pairs of input sequences. Moreover, Fried et al. (2018), Jain et al. (2019), and Zhu Y. et al. (2020) attempted some data augmentation techniques to optimize the effect of the R2R datasets in the model training. For outdoor navigation, Karl et al. (2020) proposed a challenging new RL environment called StreetNav, based on Google Street View, consisting of natural images of real-world locations and realistic connections between them. Nowadays, how to better perceive multi-modal information and achieve matching and aligning of vision and language information has been one of the research hotspots in this field (Lianbo et al., 2021c).

Considering many scholars have proposed different multi-modal fusion methods (Fried et al., 2018; Landi et al., 2019; Hwang and Kim, 2021), it still can be improved. There are two main reasons as follows: (1) The recent fusion methods cannot accurately work on the relatively important features of visual and textual information, including landmark objects under the scene and landmark directions under the instructions; (2) Facing with complex indoor environment, the internal connections among pairs of perceptual inputs cannot reflect a large amount of useful and helpful information, when these connections are ignored. To this end, we propose a novel model of the key information matching network, VIM-Net, which employs multi-modal matching methods and navigation self-tuning modules. In the detailed design of the model, VIM-Net model principally consists of two matching modules, visual matching module(V-mat) and trajectory matching module (T-mat). Specifically, the main task of V-mat is to match the target object information, visually captured from camera, with entities information extracted from the instructions. The main task of T-mat is to match the motion information identified in the trajectory with the direction information extracted from the instructions, and then the machine can compare the difference between the trajectory of target object and the actual path. If the score of the matching path mechanism is below the threshold, it will be corrected and forced to return to the previous position. In addition, the traditional processing method only aims at enhancing the matching effect by increasing the amount of datasets, but the characteristic information of datasets itself is ignored. To solve this problem, the VIM-Net model enhances the global feature fusion on the basis of original visual and linguistic information. Therefore, combined with the inspiration of human self-positioning perception, we approach this problem from two aspects. On the one hand, these paths generated by the action predictor are sampled as the third type of data information to match text instructions. On the other hand, those parameters are retrained and adjusted by adopting the heuristic self-tuning module to plan a more reliable path, thereby prompting the increasing accuracy of the specified navigation.

Overall, our model cannot only strengthen the fusion understanding of multi-modal information, but also be conducive to multi-modal tasks in other domains, such as visual question answering (VQA) (Antol et al., 2015), image or video caption generation (Das et al., 2017). Our contributions mainly include:

(1) We proposed a new neural network model, VIM-Net, which combines image recognition information from objection detection methods and extracts linguistic entities from natural language processing methods, to optimize the utilization of the datasets;
(2) The generated path and the language instruction can be matched and aligned at the control of the self-tuning module based on heuristic, which retrains parameters at the more appropriate position to optimize the path planning and improve the robustness of the network model;
(3) Validation experiments are carried out on the R2R and RUN datasets, and the results are compared with the baseline results. Virtually, the experimental results show the VIM-Net model can greatly improve the navigation accuracy of VLN tasks.

## 2. Motivation

In this section, we first explain that it is universal to ignore the object matching of vision and language features. Based on the research gaps identified, we present an outline of our proposed approach.

### 2.1. Current research

**Figure 1:**
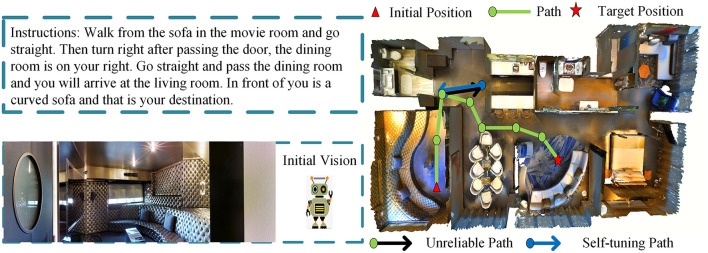
Examples of vision and language navigation (VLN) tasks are executed by VIM agent.

As Figure 1 illustrates, perceptual vision contains a large amount of environmental information (Huang et al., 2019), such as various tables, decorations, room layout, etc. Changeable natural language instructions require agents to perform compound actions (Majumdar et al., 2020), such as “turn right after passing the door, the dining room is on your right” and “go straight and pass the dining room and you will arrive at the living room”. Therefore, to reach a destination accurately, it is of great significance for the VLN agent to discover connections among multiple modalities. In particular, three steps should be performed. Step 1: locate the agent and determine the next action; step 2: identify landmarks from perceptual vision; and step 3: choose the single action to implement the compound action in accordance with instructions.

Additionally, the agent should be able to make corresponding actions according to multi-modal features, which are derived from data fusion based on the natural language instructions and real-time vision (Qi et al., 2020). However, previous work focused on increasing the amount of input data and ignoring the matching between the target information and vital information within the instructions. Given the large proportion of useless characteristics, it is highly possible to generate unreliable paths (Ma et al., 2019). According to the datasets, each complete track is obtained by serializing a small track that generated trajectory far from the original predetermined route, thus resulting in poor navigation accuracy.

### 2.2. Research gaps

**Figure 2:**
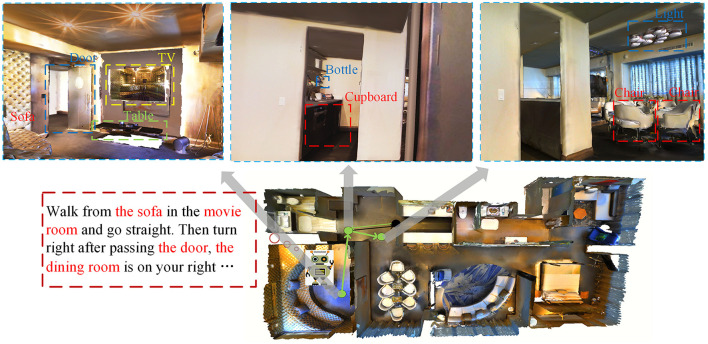
Examples of the keywords of instructions and the target features of vision have certain corresponding relationship.

In response to the above-mentioned situation, we found that the detailed matching information between perceptual vision, language instructions, and serialized trajectories is often ignored. This phenomenon is not only in the vision-and-language navigation, but also in other research fields, such as dialogue navigation, outdoor scene navigation, etc. (Chen et al., 2019; Nguyen and Daumé, 2019; Yan et al., 2019). However, features can be extracted and fused in some cases. As shown in Figure 2, we extracted keywords from instructions, such as TV, sofa, movie room, etc. These keywords will be matched with the target features extracted from the vision without considering the useless features that account for a large proportion of instructions, thereby avoiding unreliable paths. The first scene can correspond to the location of the movie room. Since there is a right-turning action, we have no way to judge the right-turning angle well. We need to compare the confidence of the second and third perspectives, which is more suitable for the dining room of instructions. If the correct direction is not selected at the beginning, we provide a self-tuning module so that the agent can return to the previous position to retrain parameters and then make a new choice. The rest of the conjunctions, etc., have no corresponding goals in the vision, and the most vital keywords occupy a relatively small proportion in the entire text, which will not play a leading role in the entire model (Hao et al., 2020).

### 2.3. Proposed method overview

Based on the above, we consider that fusion matching based on the target features of the perceptual vision and the keywords of the language instruction can improve the multi-modal matching ability and optimize the limitations of increasing the amount of datasets to improve accuracy. This method makes full use of the subtle connections between the two modalities, grasping the impact of vital information in the entire scene, and greatly optimizing the data input quality of the tasks. Not only that, we also explored the potential connection between the instructions and trajectories, and used the self-correction module to correct deviations in navigation. Considering this phenomenon, we propose the VIM-Net network, a multi-modal data fusion model based on vital information matching, to solve the problem that existing methods cannot make full use of internal characteristics of the datasets.

## 3. Related work

To improve the accuracy of visual language navigation tasks, many scholars (Fried et al., 2018; Ma et al., 2019; Wang et al., 2019; Majumdar et al., 2020; Zhu F. et al., 2020; Hwang and Kim, 2021; Lianbo et al., 2021a,b) have made lots of contribution. The Speaker–Follower model is proposed by Fried. The model is mainly divided into two modules: Speaker module and Follower module. The Speaker module outputs the corresponding language label according to the path, and the Follower module is responsible for outputting the path according to the input text command. However, the model proposed by the author still ignores the Vital Information of the entire navigation task, that is, typical landmark objects or obvious location words, and the error between the real trajectory and the text instruction, which leads to the effect of the entire model is still not ideal. Therefore, this paper proposes the VIM-Net model. In the network, we propose two main modules, the visual instruction matching module and the trajectory instruction matching module. The detailed information under the navigation task is deeply excavated, and this method fundamentally solves the above-mentioned problems.

## 4. Model design

**Figure 3:**
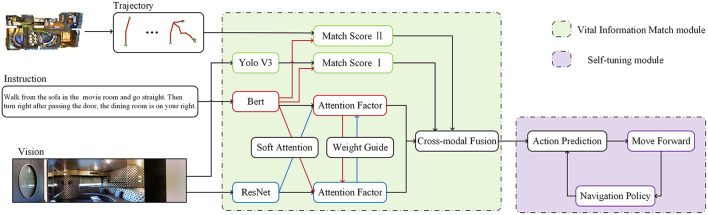
Schema of the proposed architecture for VLN. The input instruction is vision, instruction, and trajectory. The VIM-Net consists of a Vital Information match module, a cross-model guide module, and a self-tuning module.

We introduce the VIM-Net in detail, which matches the information after image target recognition with the features extracted from language entities. The modal contains two modules: the vision-instruction matching module and the trajectory instruction matching module as illustrated in Figure 3. In the vision-instruction matching module, we use the Yolo algorithm to extract the object features after target recognition and the features processed by the entity extraction component of instructions, compare them and input them into the action prediction module to guide the generation of navigation actions (Redmon et al., 2016).

To further improve the overall integrity of the navigation, we introduce a self-correcting trajectory module that matches the path and the instruction. In this module, we refer to the feedback ideas of Ke et al. (2019), improve the relevant evaluation score indicators, and increase the robustness of the entire system.

### 4.1. The vision and instruction matching module

In view of the fact that traditional processing methods only increase the matching effect by increasing the volume of data, replicating the datasets, or simply complicate the datasets (delete and modify the datasets or add other types of data), these methods still ignore the characteristics of the data set itself. Therefore, our visual instruction matching module is used to determine whether the agent has reached the landmark of the predetermined trajectory, which is the key matching part of the two types of information (Vasudevan et al., 2021). The image information obtained by the concrete vision is matched with the entity extracted from the language input. In the original visual and language information, the matching of entity information in the data stream is focused on, thereby improving the overall multi-modal matching ability (Zhao et al., 2021).

For panoramic images, the orientation features based on the pre-trained Reset-152 along with the agent, will be input into the visual features of the convolutional neural network (CNN). The instruction embedding datasets for each mode is input as the joint multi-modal embedding module so that embedding features can be generated based on intermodal data exchange. We propose to input the perceptual visual features processed by Yolo and the instruction keywords extracted by the entity extraction component to the visual instruction matching module, and set the controller of the scoring module as:


(1) $$\begin{array}{l} {\text{Ψ}}_{t}\left(s_{t},h_{t-1}\right)\in \left(0,1\right) \end{array}$$

where the value is 1, it means that the agent has reached the aimed landmark, and 0 means that it has not. The controller is trained to make decisions at variable time steps by Ψ_*t*_, which is s an Adaptive Computation Time (ACT) LSTM. The hidden state of the controller is represented by *h*_*t*−1_. In the Visual and instruction matching work, Ψ_*t*_ uses the variable number of intermediate navigation steps to identify the landmarks.

### 4.2. Track and instruction matching module

The inspiration for the trajectory instruction matching comes from the fact that when people find their way, they will confirm whether they have not deviated from the trajectory when they reach a landmark or need to turn. Some scholars have made bold attempts before. MT module is used as external memory by Gordon and others to remember clearly the traversal path of the agent from the newest visited landmark (Vasudevan et al., 2021). Then, the MT module is reinitialized once the time that the agent is nearest or on the iconic location, where the most recently visited landmark information is stored after the memory MT have been reinitialized. In this way of re-initialization, the relevant direction indication is better located and matched with the help of the trajectory instruction matching module.

**Figure 4:**
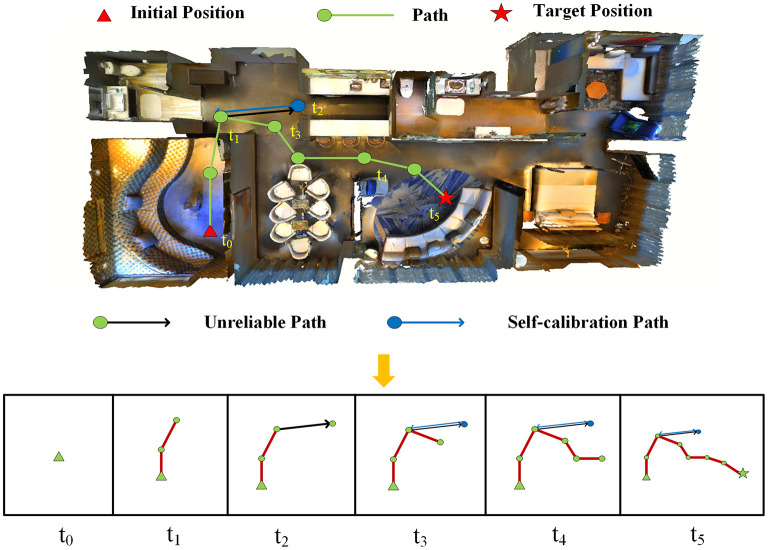
Examples of navigation trajectory. The path is rasterized and written into the memory image.

As Figure 4 shows, writing module is set up to write down the traversed path into the memory and compute it in the simulation system. The path from the most recently visited key points to the current location is tracked, rasterized, and written down into the memory image. In this image, the red line represents the path, and the blue square marks the starting point. To ensure space in all direction be stored, the center of the memory image is always saved into the writing module. If the image size is exceeded from a new rasterized pixel of the coordinates, the proportion of the stored image is increased until the new pixel can hold the image.

By recording the characteristics of the trajectory and inputting the corresponding instructions to the trajectory instruction matching module, the controller of the scoring module is set as:


(2) $$\begin{array}{l} \varphi_{t}\left(m_{t},h_{t-1}\right)\in \left(0,1\right) \end{array}$$

where the value is 1, it means the aimed landmark has been reached, and otherwise, it is 0. The controller is trained to make decisions at variable time steps by φ_*t*_, which is an Adaptive Computation Time (ACT) LSTM. The hidden state of the controller is represented by *h*_*t*−1_. In the Visual and instruction matching work, φ_*t*_ uses the variable number of intermediate navigation steps to identify the landmarks.

### 4.3. Action prediction module

The action at time *T* is defined as the weighted average of Ψ_*t*_ and φ_*t*_. For different parts of the trajectory, the input of the action predictor mainly depends on two inputs λΨ_*t*_+φ_*t*_. For example, when the next Vital Information is not visible, the prediction should rely on φ_*t*_; when the Vital Information is clearly identifiable, both inputs are input to the predictor. After the final data analysis, we determined that when λ= 0.75, the overall network effect is optimal. The learned matching score will adaptively decide which predictions are trustworthy and how many are passed in each step. This adaptive fusion can be understood as a calibration system of two complementary subsystems for motion prediction. Whether to perform corrective action requires time and space to adjudicate. In this case, the input of the action predictor is enriched, more accurate navigation actions are trained, and then the navigation trajectory with less error is serialized.

### 4.4. Review of the target detection component

As algorithms for object detection have made important advances, the R-CNN algorithm and one-stage algorithms have become popular. The R-CNN algorithm bases on a region proposal (Fast R-CNN and Faster R-CNN). They are two-stage and generate region proposals using heuristic methods or CNN networks, and then perform classification and regression on the region proposals (Girshick, 2015; Mao et al., 2019). The other one-stage algorithms predict the categories and positions of different targets using a CNN network merely. In terms of accuracy, the first algorithm has more advantages than the second algorithm, and in terms of speed, the second algorithm is better.

**Figure 5:**
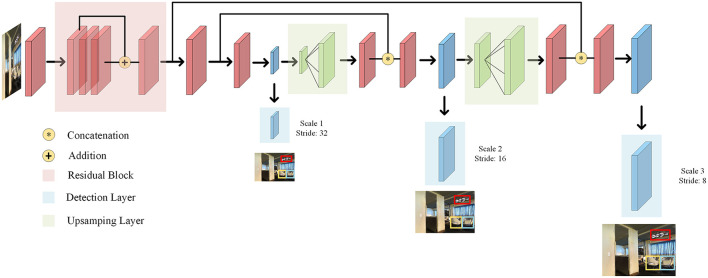
Scheme of the proposed architecture for Yolo.

As illustrated in Figure 5, we use the Yolo algorithm, which is detected by a CNN network. It is a single-pipe strategy, and its training and prediction are both end-to-end, so the Yolo algorithm is relatively simple and fast. Then, since Yolo convolves the entire picture, it has a larger field of view in the detection target, and it is not easy to misjudge the background.

### 4.5. Review of entity abstraction component

**Figure 6:**
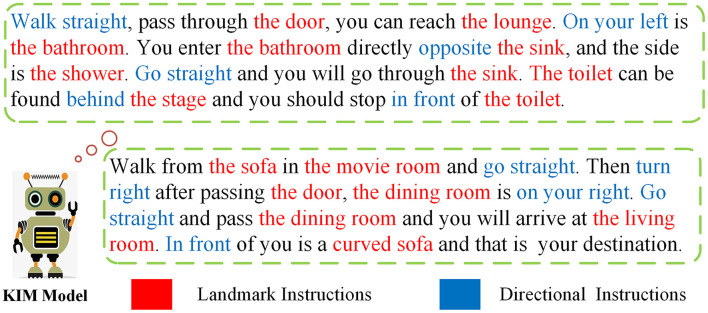
Examples of instruction for navigation. We use entity abstract components to extract different types of subjects (streets, restaurants, etc.) and number them in the order of occurrence of sentences.

To further consider the degree of matching between the key target information and the instruction information, we introduced entity abstract components to process the characteristic information under the instruction information. We adopt a similar approach to Suhr et al. (2018), replacing phrases in the sentences, which refer to previously unseen entities with variables. E.g., “Walk from kitchen to sofa” turns into “Walk from X1 to Y1”. Here, X1 and Y1 are the feature points that match the perceived visual information. We use entity abstract components to extract different types of subjects (streets, restaurants, etc.) and number them in the order of occurrence of sentences (Paz-Argaman and Tsarfaty, 2019). As illustrated in Figure 6, the number is reset after each instruction is completed. Hence, the model contains only a small number of Vital Information, such that the matching efficiency of the overall model is significantly improved (Iyer et al., 2017). We use an encoder–decoder model with global attention, where the anonymized utterance is encoded using a bidirectional LSTM network.


(3) $$\begin{array}{l} c_{i}=\overset{k}{\underset{j=1}{\sum }}\alpha_{i,j}\cdot s_{j} \end{array}$$

(4) $$\alpha_{i,j}=\frac{exp(h_{i}^{T}Fs_{j})}{\sum_{j=1}^{k}exp(h_{i}^{T}Fs_{j})}$$

(5) $$\begin{array}{l} h_{i},m_{i}=f\left(h_{i-1},m_{i-1},c_{i-1}\right) \end{array}$$

α_*i, j*_ is the attention weights which are the results of an inner product between the decoder hidden state for the current time step *h*_*i*_ and the hidden representation of the source token *s*_*j*_. *F* indicates linear transformation. The next hidden state *h*_*i*_ and cell state *m*_*i*_ are computed by the decoder LSTM cell *f* based on the previous hidden and cell states *h*_*i*−1_,*m*_*i*−1_ and the context vector of the previous time step *c*_*i*−1_.

### 4.6. Learning detail

In our model, the student-forcing method is followed under the training of supervised manner. In every step the motion prediction module is trained by the signal attached to motion with direction of the next landmark from monitor. For loss function, we select cross-entropy loss function to evaluate the effect of action module and the matching module after training. Moreover, cross-entropy loss function is normally applied to classification tasks. The total loss is from the sum of all modules:


(6) $$\begin{array}{l} {Loss}_{all}={Loss}_{vision-matching}+{Loss}_{track-matching}+{Loss}_{action} \end{array}$$

The loss of the two matching modules only takes effect at the landmarks of the landmarks, and these landmarks are more than the road nodes for calculating the motion loss and trajectory error loss. Therefore, we first train the matching networks separately for the matching task, and then integrate them with other components into the overall training. We use *Loss*_*all*_ to train the entire network.

## 5. Experiments

Our experiments focus on (1) determining the parameter configuration, including the training cycle of the entire neural network and the value λ in the formula *val* = λΨ_*t*_+φ_*t*_ in the VIM-Net model; (2) multiple ablation research to further explain our model; (3) applying our model to other datasets to evaluate our model.

### 5.1. Experimental settings

#### 5.1.1. Datasets

The Room-to-Room(R2R) vision-and-language navigation datasets are used as experimental evaluation (Anderson et al., 2018). First, a human-generated navigation instruction which describes the path to the destination can be provided by the agent at one certain location. Then, multiple discrete actions, including turning, moving, etc., will be followed and carried out to navigate to the global location. Finally, the agent execute “stop” instruction to come to an end. Some robotic navigation settings, it should be noted, are different from the normal. On the one hand, the agent is not provided with the global images, but have to figure out whether it reaches to the goal or not from the text description and environmental information. On the other hand, the Matter3D navigation maps, where each path consists of 5–7 discrete viewpoints and its physical length approaches to 10 m, are applied into this system. The Matterport3D Simulator is a large-scale visual reinforcement learning (RL) simulation environment for developing intelligent robots based on the Matterport3D datasets. It allows concrete robots to virtually “move” across the scene by adopting poses consistent with the panoramic viewpoint. For each scene, the simulator contains a weighted undirected graph over the panoramic viewpoint. The presence of an edge thus represents a robot navigable transition between two viewpoints. And from one viewpoint to another, movement follows any edge in the navigation graph. The simulator does not define or constrain the agent's goals, reward functions, or any other context. Moreover, there are 21.5k artificial instructions in total, three of which are given to each path, about 29 words per instruction averagely. The datasets are split into training, validation, and test set. Especially, the validation set is split into two parts: (1) routes sampled from environmental perception with the camera during the training(seen); (2) environments information that has not been seen during the training(unseen). All the test set routes come from new environment except the environment from the training and validation set.

The input of the Run task consists of three parts. One is a very detailed map, which is divided into many blocks. The other is a clear starting point. The third is a navigation sequence. The navigation sequence contains many navigation instructions, which will be carried out one by one in order. The agent will reach the preset destination according to the guidance of these instructions. The Run task will output the entire navigation route, including the coordinates of fixed points on the map. The Run datasets are designed and collected for run tasks. It collects three densely populated areas in downtown Manhattan, covering an area of 0.5 square kilometers, including 2,515 instructions and corresponding routes. These natural language navigation instructions are aligned with OpenStreetMap (OSM) based coordinate matching (Paz-Argaman and Tsarfaty, 2019).

#### 5.1.2. Evaluation metrics

Our main evaluation metrics are navigation bias, success rate, navigation path length, success rate at any point, and weighted success rate for reverse path length. The NE result is expressed as the average distance between the agent's final arrival point and the target location. SR represents the percentage of the agent's final position that is less than 3 m from the target position. The result of SPL is expressed as the total length of the predicted path. It is definitely optimal when it is equal to the length of the reference path. OSR is expressed as a measure of the frequency with which any node in the path is within 3 m of the target, allowing the agent to overtake the target without penalty, which is represented by the last node of the reference path. The success of SPL weighting takes into account the success rate and the path length. Although it objectively evaluates the execution effect of intelligent robots to a large extent, it does not consider the similarity between the intermediate nodes of the predicted path and the reference path. This poses a problem, even though it shows a high score, the predicted path does not really follow the verbal instructions, it just hits the correct goal. The formula for calculating SPL is as follows:


(7) $$\begin{array}{l} SPL=\frac{1}{N}\overset{N}{\underset{i=1}{\sum }}S_{i}\frac{L_{i}}{max\left(P_{i},L_{i}\right)} \end{array}$$

where N represents the count of episodes, *S*_*i*_ is the success indicator under the binary of the episode (whether it is successful or not), *P*_*i*_ and *L*_*i*_ represent the actual path length and theoretical shortest path distance under the episode.

Because visual language navigation is a very challenging task, it is not possible to rely on a single indicator to measure the quality of the overall task. It must be ensured that the corresponding actions can be made according to the instructions throughout the navigation process instead of only observing the starting and ending points and evaluating the quality of the model in general terms. When we analyzed the results, we fully considered this situation, and tested different indicators respectively, and strived to objectively evaluate our models in different dimensions.

#### 5.1.3. Implementation details

The experiments in this paper use the provided server Nvidia GTX TitanXP GPU, the development environment is the operating system Ubuntu 16.04, and the mainstream deep learning framework used is the Pytorch framework. We generate dynamic filters with 512 channels using a linear layer with dropout (*p* = 0.5). In our attention module, q and K have 128 channels and we apply a ReLU non-linearity after the linear transformation. For our action selection, we apply dropout with *p* = 0.5 to the policy hidden state before feeding it to the linear layer. We use the vision feature vectors from the convolutional layers in the way of ResNet training on the ImageNet classification datasets. But the problem is the ReNet cannot be updated because these features are changeless after the final training. For better optimizations, BERT is used to initialize word-embedding vectors in the process of experiment. Also, dynamic filters with 512 channels with a linear layer combined with dropout (*p* = 0.5) are generated.

### 5.2. Experimental parameter configuration

**Figure 7:**
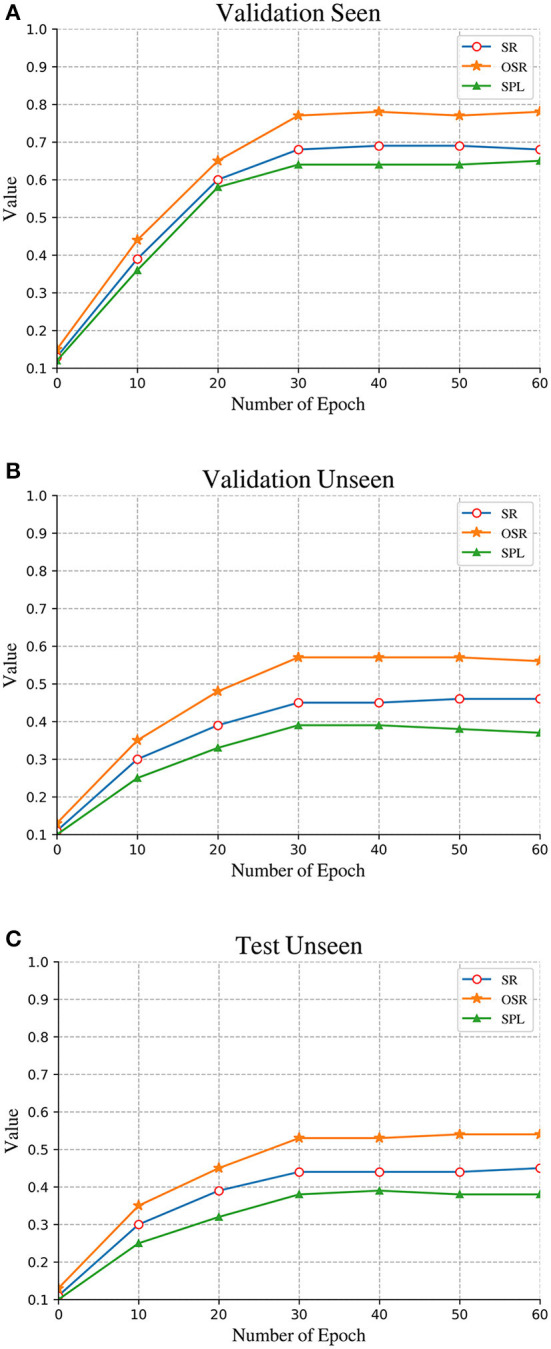
Comparison of performance on the different condition: **(A)** Validation seen. **(B)** Validation unseen. **(C)** Test unseen.

As shown in Figure 7 and Table 1, We compared the changes in the accuracy rate of different training cycles, and found that when the Epoch is 30, the basic model has completed the convergence, and the fluctuation of various indicators is not obvious. Although not all indicators can reach the peak when the Epoch is 30, considering the training efficiency of the entire model, the overall training can be ended when there is no significant improvement. In view of the reason why only an integer number of cycles are selected for training, the main reason is that each instruction needs to be trained due to the constraints of the datasets. The computing resources are very large, and it is difficult to make accurate judgments. Compared with the existing models, it is found that most models will converge in a short period to avoid over fitting.

**Table 1:** The evaluation index results table.

**Epoch**	**Validation seen**				**Validation unseen**				**Test unseen**			
	**SR↑**	**NE↓**	**OSR↑**	**SPL↑**	**SR↑**	**NE↓**	**OSR↑**	**SPL↑**	**SR↑**	**NE↓**	**OSR↑**	**SPL↑**
0	0.13	-	0.15	0.12	0.11	-	0.13	0.10	0.11	-	0.13	0.11
10	0.39	4.12	0.44	0.36	0.30	6.91	0.35	0.25	0.30	8.36	0.35	0.25
20	0.60	3.79	0.65	0.58	0.39	6.09	0.48	0.33	0.39	7.14	0.45	0.32
**30**	**0.68**	**3.23**	**0.77**	**0.64**	**0.45**	**5.63**	**0.57**	**0.39**	**0.44**	**5.76**	**0.53**	**0.38**
40	0.69	3.19	0.78	0.64	0.45	5.57	0.57	0.39	0.44	5.70	0.53	0.39
50	0.69	3.15	0.77	0.64	0.46	5.40	0.57	0.38	0.44	5.55	0.54	0.38
60	0.68	3.15	0.78	0.65	0.46	5.40	0.56	0.37	0.45	5.56	0.54	0.38

When the Epoch is 30, each index has reached a relatively high level.

**Figure 8:**
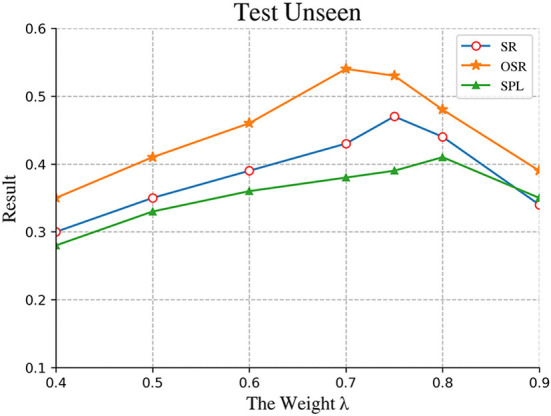
Comparison of performance on the different weight of scoring module. The best result is approximately obtained when the is equal to 0.75.

We compared different results and plotted images to further observe the changes in the results of different training epochs. As shown in Figure 8 and Table 2, When λ was equal to 0.75 in the formula *val* = λΨ_*t*_+φ_*t*_ in the VIM-net model, the effect of the entire model was optimal. Observing the changes of the two variables Ψ_*t*_ and φ_*t*_ in the formula, it is found that φ_*t*_ represents the score of the T-mat module, and its values are generally divided into high-grade and low-grade. The high-grade is about 0.8 and above, and the low-grade is about 0.3 and below. The main influencing factor is whether the planned path is consistent with the direction in the instruction. The value of Ψ_*t*_ fluctuates more obviously, indicating that the V-mat module compares whether there are targets consistent with the command in the current field of view. Since each command does not necessarily have only one target, the Ψ_*t*_ score is 1 when all targets are found, that is, the overall score is low, and the influence value is weaker after multiplying the coefficient λ.

**Table 2:** Comparison of the test unseen set.

**The weight λ**	**Test unseen**			
	**SR↑**	**NE↓**	**OSR↑**	**SPL↑**
0.4	0.30	6.57	0.35	0.28
0.5	0.35	6.39	0.41	0.33
0.6	0.39	6.15	0.46	0.36
0.7	0.42	5.87	**0.55**	0.37
0.75	**0.44**	**5.76**	0.53	**0.39**
0.8	0.41	5.89	0.48	0.37
0.9	0.36	6.32	0.39	0.35

When the value of λ is 0.75, each index has reached a relatively high level.

It is very important to consider the overall value of the path with high confidence. After a lot of experiments, it is found that if the value of *Val* is selected higher, the training period of the entire network will be longer, and the growth of each index will not be significant. However, paths with too low confidence have little improvement on the original network, which is not enough to support the needs of the VIM-Net network. Therefore, after comparative experiments, it is found that the value of about 1 has well-coordinated the balance between accuracy and efficiency.

### 5.3. Ablation study

We test the impact of our implementation choices on VLN in our ablation study and the results are presented in Tables 3, 4. First, we compared the VIM-Net model with a model that uses a simple replication data set to expand the current data set to determine the impact of the Vital Information matching module on the entire network. Then, we introduced the importance of using the trajectory self-tuning module to correct the entire navigation task. Finally, we performed ablation experiments on the RUN datasets and compared them with CGAEW model (Paz-Argaman and Tsarfaty, 2019) and the experimental results are shown in the Table 4.

**Table 3:** The ablation study of our proposed architecture on the R2R validation set.

**Method**	**Num**	**V-mat**	**T-mat**	**Self-tuning**	**Validation seen**			
					**SR↑**	**NE↓**	**OSR↑**	**SPL↑**
Speaker-Follower					0.63	3.4	0.71	-
VIM-Net	1	√			0.67	**3.16**	0.74	0.59
	2		√		0.64	3.26	0.76	0.62
	3			√	0.66	3.61	**0.71**	0.55
	4	√	√	√	**0.68**	3.23	**0.77**	**0.64**

This is the result of ablation experiment. The effect of all VIM-Net is better than that of single module.

**Table 4:** The ablation study of our proposed architecture on the R2R validation set and our baseline model is the Speaker-Q14 Follower model.

**Model**	**Validation Unseen**				**Test Seen**			
	**SR↑**	**NE↓**	**OSR↑**	**SPL↑**	**SR↑**	**NE↓**	**OSR↑**	**SPL↑**
Speaker-Follower	0.38	6.68	0.42	-	0.36	6.69	0.42	0.28
T-mat	0.44	6.03	0.54	0.38	0.43	5.97	0.45	0.34
V-mat	0.43	5.34	0.50	0.36	0.41	5.59	0.54	0.37
Self-tuning	0.41	6.79	0.56	0.32	0.39	6.81	0.46	0.31
VIM-Net	**0.45**	5.63	**0.57**	**0.39**	**0.44**	5.76	**0.53**	**0.39**

#### 5.3.1. V-mat module

As the results show, the performance in datasets processing well outperforms traditional VLN data augmentation methods. This is because the model can more accurately determine the intrinsic connection between the perceptual visual and textual instructions under the input datasets. Our V-mat visual matching module improves the success rate by 3% compared to the baseline model using the pure replication datasets. Not only that, we observed the indicators of SR and OSR and found that the improvement of the two is obviously different. After analysis, it can be roughly concluded that the original model can also match some information of the command to calibrate the path during the navigation process, but for the end point. Obviously, the accuracy of SR cannot reach our model, which explains that the improvement of SR is obvious when the improvement of OSR is not obvious.

#### 5.3.2. T-mat module

The improvement effect of the T-mat module on the entire task is not particularly obvious. Although there is a small improvement, it does not have a good improvement for a certain indicator. After our analysis, it is found that this is because this module aims to discover whether the planned trajectory is consistent with the navigation instructions, and focuses on whether the direction conversion is reasonable. Compared with the T-mat model, which inherently matches key target information, our T-mat trajectory matching module focuses more on whether the connection between multi-segment instructions is correct, so the accuracy is increased by 3%. Compared with all the indicators, we found that SPL has the largest improvement. This indicator is reflected in the ratio of the success rate and the path length, which better judges the module's overall grasp of the planning process, and ensures that the agent can be planned from a macro perspective path quality.

#### 5.3.3. Self-tuning module

Our method is significantly better than the self-monitoring agent using greedy decoding. When the progress marker can use the features of each navigable direction previously accessed, but the trajectory self-tuning module is not available, the performance will not increase significantly. However, the use of the gated attention mechanism is good for extracting the overlap between the position of the landmark under the text instruction and the real trajectory, which means that the network can use this information to improve action selection. Compared with the baseline model that uses pure soft attention to compare the error of the entire trajectory, our method can achieve a moderate gain, which reflects the purpose of intelligent navigation.

Through the above analysis, compared with the original Speaker–Follower model, our three modules are indeed of great help in improving the accuracy of the original task from different angles, although looking at a module alone may sacrifice some indicators, but the comprehensive consideration of VIM-Net undoubtedly achieves the desired effect. Not only that, we also compare the VIM-Net model with some existing models, and the results are shown in Table 5.

**Table 5:** The display table of results from other models.

**Model**	**Validation unseen**				**Test seen**				**Test seen**			
	**SR**	**NE**	**OSR**	**SPL**	**SR**	**NE**	**OSR**	**SPL**	**SR**	**NE**	**OSR**	**SPL**
Random	0.16	9.45	0.21	-	0.16	9.23	0.22	-	0.13	9.77	0.18	0.12
Student-forcing	0.39	6.01	0.53	-	0.22	7.81	0.28	-	0.20	7.85	0.27	0.18
RPA	0.43	5.56	0.53	-	0.25	7.65	0.32	-	0.25	7.53	0.33	0.23
DynamicConv-agent	0.53	4.68	0.66	0.46	0.32	6.65	0.44	0.27	0.31	7.14	0.42	0.27
Seq2Seq	0.53	4.68	0.66	0.46	0.32	6.65	0.44	0.27	0.31	7.14	0.42	0.27
Speaker-follower	0.63	3.40	0.71	-	0.38	6.68	0.42	-	0.36	6.69	0.42	0.28
VIM-Net	0.68	3.23	0.77	0.64	0.45	5.63	0.57	0.39	0.43	5.76	0.53	0.38
Regetful	0.68	3.31	0.77	0.63	0.50	5.32	0.59	0.41	0.48	5.69	0.56	0.40
Regetful+VIM-Net	0.70	3.20	0.79	0.65	0.50	5.10	0.61	0.42	0.49	5.53	0.55	0.41

#### 5.3.4. Comparison with other models

As shown in Table 5, We found that the proposed VIM-Net model performs well, not only in the Speaker–Follower model but also when we migrate its V-mat and T-mat modules to the Regretful model, although not all the indicators have all increased by a large margin, but it can be guaranteed that some indicators have steadily increased, and no indicators have fallen significantly. We also compared the Random model, the Student-Forcing model and the RPA model. After comparing different models and integrating different modules, we found that the modules we proposed are basically applicable to all models to varying degrees, which indeed affirms our work. Comparing the indicators of the modified Seq2Seq network still cannot reach the VIM-Net network that integrates advanced algorithms, the main reason is that the general model based on the encoder-decoder network cannot more accurately find the overall network input. Therefore, the latter multi-modal fusion module based on dynamic filter cannot realize the alignment and fusion between modalities. However, the research significance of the Seq2Seq network is reflected in the proposal of a general and easy-to-transfer framework to solve many multi-modal tasks, which is convenient for the development of related topics later.

#### 5.3.5. Results of the RUN datasets

We follow the evaluation method of Paz-Argaman and Tsarfaty (2019). For the model to be evaluated, we measure whether the navigation process is successful when the Euclidean distance between the agent's location and the destination is within 5 tile, and the agent should also face the right direction. For each instruction, we will extract the corresponding entity from the map and move it. As shown in Table 6, from the experimental results, the result of our T-mat is better than that of CGEAW, because our T-mat focuses on the connection between instruction sequences, which can improve the navigation accuracy. Self tuning module will ensure the confidence of each navigation path. Although some path length indicators will be sacrificed, the overall accuracy will be improved significantly. Compared with CGEAW model, the accuracy of VIM-Net model has been improved by more than 30%. This shows that the important information matching proposed by us is helpful to solve the navigation problem. Considering that the RUN datasets is built in an outdoor scenario, it shows that VIM-Net performs well for indoor and outdoor navigation, which confirms our work.

**Table 6:** The ablation study of our proposed architecture on the RUN datasets and comparison with other models.

**Model**	**SR↑ (%)**
CGAEW	10.45
V-mat	-
T-mat	35.46
Self-tuning	28.98
VIM-Net	44.31
VIM-Net+CGAEW (attention layer)	47.60
Human	81.12

## 6. Conclusion

Conventional navigation system pays more attention to the construction of the global scene in the map before it works, and marking the initial position and destination position. Generally, with the help of beam search or greedy algorithm, the most suitable trajectory is derived, based on deep learning visual language navigation focusing on vision and text. Then, the next navigation behavior can be deduced, in which vision information and text information will be independently processed, and each unit is applied by the more mature algorithms in the field. After these, multi-modal information will be simply aligned and spliced to judge and decide the next navigation behavior, under supervision learning. Undoubtedly, this way of work procedure can efficiently save lobar and time costs. Consequently, Our research proposes a new type of deep neural network, VIM-Net model, as an effective tool to deal with VLN tasks. It aims to make best use of temporal information and multi-modal fusion information extracted with the joint multi-modal embedding module. Besides, VIM-Net can increase success rate and search efficiency of the task, on the basis of backtracking function from optimal greedy local search algorithm. Finally, we carried out various experimental validations on the R2R and RUN datasets and the results demonstrated the superiority of our model.

This paper has carried out rich research on the subject of visual language navigation. Through the analysis of different networks and modules, a relatively optimized experimental model is proposed, and the ideal effect is achieved. However, there are still many places worth studying in the task itself, and follow-up research will focus on the following aspects.

(1) Validation on different datasets. Compared with the mainstream Room to Room datasets, other newly proposed datasets, including Room for Room and TouchDown in outdoor scenes, all have great challenges. Whether the proposed model is generalizable and general for different environments and different tasks still needs to be considered. If there is such a deficiency, what kind of optimization can be reasonably adapted to all tasks. (2) Further enhance the effect of the entire network. The network proposed in this paper still contains many inexplicable parts. In the overall selection and parameter adjustment, we still cannot find a general and reasonable way to perform the required purpose. And in the selection of many hyperparameters, we still have to waste a lot of time and computing resources to select the most suitable value. In the future work, the reusability and parameter sharing among various modules of the network can be further considered. (3) Optimize the real-time and practicality of the entire network. The current work still spends a lot of time to compare the accuracy of the training data set and the validation data set, and the calculation time of the overall network is still not negligible. Second, the training in the simulator ignores factors such as lighting and dynamic complex scenes in the real environment, and future work can try to reproduce it on robots.

## Author contributions

ZJ and JR designed the model. SY and KY conducted the experiments and wrote the papers. SC polished the paper. All authors contributed to the article and approved the submitted version.

## Funding

This research was funded by the National Natural Science Foundation of China (61872073), the Fundamental Research Funds for the Central Universities (N2126005 and N2126002), and the National Natural Science Foundation of Liaoning (2021-MS-101).

## Conflict of interest

The authors declare that the research was conducted in the absence of any commercial or financial relationships that could be construed as a potential conflict of interest.

## Publisher's note

All claims expressed in this article are solely those of the authors and do not necessarily represent those of their affiliated organizations, or those of the publisher, the editors and the reviewers. Any product that may be evaluated in this article, or claim that may be made by its manufacturer, is not guaranteed or endorsed by the publisher.
