# Supplementary material for: Vital information matching in vision-and-language navigation
Source: Front Neurorobot. 2022 Nov 17;16:1035921. doi: 10.3389/fnbot.2022.1035921 (PMC9712967; doi:10.3389/fnbot.2022.1035921)
Supplement: Supplementary file 1 [file Presentation_1.pdf]

Supplementary Material

1 SUPPLEMENTARY DATA

Download the train, val_seen, val_unseen, and test splits of the CVDN and NDH dataset by executing: sh tasks/CVDN/data/download.sh sh tasks/NDH/data/download.sh
